# Treatment of Ossific Diseases of the Joints by Hypodermic Injections*Read at the third regular meeting of the Missouri Valley Veterinary Association, at Atchison, Kan., December 12th, 1894.

**Published:** 1894-12

**Authors:** Junius H. Wattles


### TREATMENT OF OSSIFIC DISEASES OF THE JOINTS BY HYPODERMIC INJECTIONS.*

By Junius H. Wattles, D.V.S.

While attending college as a student, the question of treating bone spavin by some other method than the actual cautery, was discussed by a gentleman who was visiting the college, and myself. We reasoned that many valuable animals were disfigured by the firing iron, and that if the same result could be obtained by some other method, it would be more satisfactory to both patron and practitioner.

The question to be answered was: Can a remedy or agent of any sort be injected into an articulation, that will produce an acute artificial inflammation, with the rapid deposit of bone cells neces-

* Read at the third regular meeting of the Missouri Valley Veterinary Association, at Atchison, Kan., December 12th, 1894.

sary to form an anchylosis, that will not cause suppuration, or some other complication or condition which will be worse than the original disease? The points in question were considered by us, with all of the misgivings and fear of ridicule that a student attending college and a gentleman in practice who had never attended any veterinary institution could have, as we felt that we were guilty of encroaching upon some of the traditions of the profession. The result was, however, that we determined to put in practice the theories which were evolved at that time, and I have done so with entire satisfaction to my patrons and myself.

The condition we know as spavin is, in my opinion, identical with the condition known as osteo-arthritis in man, and nearly the same general pathology occurs in both cases; the result desired, however, is not the same, as in man motion is usually insisted upon, even in cases where the articular cartilage is destroyed, to prevent anchylosis, while in our cases we desire complete anchylosis in as short a time as possible, with the exostosis reduced to a minimum.

Looking upon the process of repair that takes place in spavin as being identical with the process of repair that takes place in the repair of a fracture of bone, and that in both cases that absolute immobility is desired for the purpose of producing anchylosis, I have used plaster of Paris, or starch bandages, and by readjusting them as the swelling subsided or they become loose from any cause, reduced the motion in a joint very materially, a plan that I have followed with advantage in those cases where it was impossible to turn the animal out to grass, after operating.

It is necessary to give a brief resumé of the general pathology of osteo-arthritis in order that my reasons for treating spavin hypodermically may be made clearer.

There is, first the injury, whatever it may be, producing in regular order congestion and inflammation, followed by exudation into the articulation, with attendant maceration and destruction of the articular cartilages. If from any cause this exudate is not absorbed, a fibrous organization takes place, preventing the union of the necessary reparative bone cells, and delaying anchylosis, but should this exudate be rapidly absorbed, it will allow the bone cells to develop faster and accumulate in a much smaller space, and thus reduce the size of the exostosis that is formed, as well as making it a compact mass, instead of the irregular porous bone tumor, with its numerous openings and channels, in which this exudate has organized and become a part of, that we usually find in these cases.

Bone spavin, ringbone, and splint, have been treated by me since March, 1886, by the hypodermic injection of tincture of iodine. My method has been as follows: I confine the animal as required; clip away the hair and wash the part with hot water; use an ordinary syringe with a large needle, inserting the needle into the tumor, usually at its upper part, and loosening up the structures with its point, withdrawing the needle slightly, and injecting about one drachm of the tincture, making several insertions of the needle in different parts of the enlargement and injecting about a drachm at each insertion, taking care not to enter large blood vessels. The patient is kept in the stable for a week or ten days, and then turned out from six weeks to three months.

A second operation is very rarely necessary, and unless the swelling becomes excessive immediately after the operation, no after treatment of any sort is required. No bad results of any kind have ever followed this method of treating this class of diseases at my hands. Complete anchylosis and termination of the lameness, in both spavin and ringbone, has always been obtained. There has never been the slightest suppuration. The enlargement does not increase materially in size, but appears to decrease when repair is in progress, by the filling in below the enlargement with new tissue. I can cite individual cases, but it is unnecessary, as you have every opportunity to test its efficacy.

The stimulant and reabsorbent action of iodine is so well known that it requires but passing mention. The beneficial results obtained by the use of iodine, hypodermically, in the cases mentioned, is the result of both actions. By the stimulation of a low grade of inflammation into an active form, it determines the rapid increase and multiplication of the reparative bone cells, and by its reabsorbent action it resolves the organized exudate into a liquified mass that is readily taken up and carried away from the part. From a general, diffused inflammation of the region, it becomes a localized inflammation that is confined to the place desired.

I do not wish to be quoted as saying that *all* cases of spavin, ringbone, or splint, can be cured by this method, but I do assert positively, that any one of these which are curable, by any other method, may be cured quicker and with less deformity or blemish remaining, by the hypodermic injection of iodine, than any other method that I have any knowledge of.

